# Notes and News

**Published:** 1900-08-11

**Authors:** 


### NOTES AND NEWS.

One of the crew of a vessel arriving at Homburg has been taken to the hospital suffering from plague.

The Queen has sent a special message of thanks to the Maharaja of Sindhai for his gift of a hospital for the use of the troops in China.

Cholera is very prevalent in India at the present time, and the interference in consequence with the despatch of troops to China continues. In Afghanistan the disease has broken out, and is spreading with great rapidity.

The new Central London Railway has several hygienic advantages over the older metropolitan lines apart from coolness and ventilation. It is much easier to keep the carriages cleaner, and consequently they are kept cleaner. The absence of rush and noise which the arrangements almost prevent is most advantageous to busy brain and overstrung nerves. It is quite surprising how quietly and leisurely it is possible to travel by the methods adopted on the new railway, which is the greatest boon in the direction of conveyance which has been introduced into the metropolis for many years.

A large number of improvements and additions have taken place recently in the hospital world. The foundation-stone of the West Ham Union Infirmary has been laid. The infirmary is to cost a quarter of a million. The buildings will occupy 20 acres, and house 672 patients. In Dumbartonshire a new fever hospital has been opened on the Kincaird Estate, for the use of East Dumbartonshire and West Stirling. The cost is estimated at £12,000 for 24 beds. In Inverness-shire the Lawson Memorial Hospital has been opened, which is a well-equipped cottage hospital, erected to the memory of the Lawsons family of Clynelish. At Coventry a new isolation ward has been added to the hospital. A new Convalescent Home for Children has been opened at Hayling Island by the Countess of Meath, and handed over to the Executive Council of the Ministering Children's League. It will accommodate 25 children, and has good grounds attached. At Romford a Passmore Edwards Hospital is being built. Colonel Burgess presented the site, and Mr. Passmore Edwards provided £4,000 for the building.

It is proposed to hold a Medical Congress in Chili, at Santiago, in December of the present year. The other South American Colonies will be represented by delegates.

Carbolic acid is now to be placed on the poison list of the Pharmacy Act. Special provision is to be made to facilitate the sale for agricultural and horticultural purposes.

We are glad to hear that immediate action is to be taken with the object of improving Netley Hospital, so as to bring it up to the level of the most efficient general hospitals of this country.

Mr. Chaplin stated in the House of Commons that the number of certificates for successful primary vaccination in 1899 exceeded those of 1898 by 169,035, which was an increase at the rate of 33·8 per cent.

The Marquis of Bute has offered £20,000 for the establishment of a Chair of Anatomy in the University of St. Andrews. In Dundee there is a feeling that the creation will prove detrimental to their own schools.

The Royal Commissioners on Hospitals in South Africa left Southampton on Saturday last for Cape Town. The "Dunottar Castle," which conveyed them, carried also 20 more nurses for the military hospitals.

Yellow fever has broken out amongst the American troops in Cuba. The camps have been removed into the country, and a strict quarantine has been established. More troops are expected, and a thorough disinfection will be effected before they arrive.

A new out-patients' department has been opened at Ancoats Hospital, Manchester, into which is incorporated accommodation for the reception of accident cases. The new department has been erected by the children of the late Mr. James Oliver as a memorial to him.

The Somerset Hospital, Cape Town, has been recently enlarged by the opening of a new Jubilee wing to accommodate 45 patients. The cost was about £13,000. A bungalow has also been added to the institution for 20 beds, and the number of patients which can now be received is 240. The Somerset Hospital is free to the poor, but a few paying patients are accepted. Government makes an annual grant to the hospital, and the Town Council votes an annual sum also for its maintenance.

Coming at this time, Mr. Fripp's letter, of July 16th, published in the *Times* on the 8th inst., must be very reassuring. He speaks of enteric abating everywhere, and the prevalence of vacant beds in the hospitals. At Bloemfontein he reports 1,600 patients, against 5,000 six weeks previously. The Yeomanry Hospital has been doing a large amount of work. The last cable reports that upwards of 980 patients had been treated. A new branch has been arranged at Pretoria. Mr. Langman has prolonged the maintenance of his hospital for another two months, as a useful work is still before it. His original offer was for six months, which time has now expired. It has been removed from Bloemfontein to Pretoria.

